# Preventive Healthcare: A Neural Network Analysis of Behavioral Habits and Chronic Diseases

**DOI:** 10.3390/healthcare5010008

**Published:** 2017-02-06

**Authors:** Viju Raghupathi, Wullianallur Raghupathi

**Affiliations:** 1 Koppelman School of Business, Brooklyn College of the City University of New York, Brooklyn, NY 11210, USA; 2 Gabelli School of Business, Fordham University, New York, NY 10058, USA; Raghupathi@fordham.edu

**Keywords:** behavioral habit, chronic disease, preventive, health care, SPSS modeler, neural network, bayesian network, association

## Abstract

The research aims to explore the association between behavioral habits and chronic diseases, and to identify a portfolio of risk factors for preventive healthcare. The data is taken from the Behavioral Risk Factor Surveillance System (BRFSS) database of the Centers for Disease Control and Prevention, for the year 2012. Using SPSS Modeler, we deploy neural networks to identify strong positive and negative associations between certain chronic diseases and behavioral habits. The data for 475,687 records from BRFS database included behavioral habit variables of consumption of soda and fruits/vegetables, alcohol, smoking, weekly working hours, and exercise; chronic disease variables of heart attack, stroke, asthma, and diabetes; and demographic variables of marital status, income, and age. Our findings indicate that with chronic conditions, behavioral habits of physical activity and fruit and vegetable consumption are negatively associated; soda, alcohol, and smoking are positively associated; and income and age are positively associated. We contribute to individual and national preventive healthcare by offering a portfolio of significant behavioral risk factors that enable individuals to make lifestyle changes and governments to frame campaigns and policies countering chronic conditions and promoting public health.

## 1. Introduction

This study emphasizes the importance of preventive healthcare for chronic diseases by identifying behavioral habits that may be linked to developing these conditions. Chronic conditions such as heart attack, cancer, chronic obstructive pulmonary disease, stroke, asthma, and diabetes are the leading causes of 70% of deaths in the U.S. [1]. These are long-lasting conditions that can be managed and controlled, although not always cured. Chronic diseases often result from unhealthy behaviors, such as lack of physical activity, poor nutrition, tobacco use, and excessive alcohol consumption, and can be prevented by introducing positive behavioral changes [1]. In the U.S., the number of people with chronic conditions has escalated over time: 125 million in 2000, 133 million in 2005, 141 million in 2010, and 149 million in 2015 [2]. By 2020, the number is expected to increase to 157 million, and by 2030 to 171 million. In terms of population percentages, the numbers represent an increase from 46.2% in 2005 to 49.2% in 2030 [3].

Providing healthcare for such a large patient population takes up 75% of the national healthcare expenditure [4]. By 2020, this figure is expected to rise to 80% [5]. The annual healthcare expenditure for a person with chronic illness is $6032, which is five times that of a healthy person ($1105). Additionally, healthcare spending for people with multiple chronic conditions amounts to more than $15,000 per annum/per beneficiary, which is roughly 15 times the amount of spending on people with no chronic conditions [2]. Most chronic diseases can be delayed, allayed, or even prevented through lifestyle changes. Chronic disease prevention and control, therefore, occupies an integral aspect of the national budget.

In the current research, we emphasize preventive healthcare for chronic diseases by focusing on the association between behavioral habits (such as smoking, alcohol consumption, fruits and vegetable consumption, and exercise) and chronic diseases (such as stroke, diabetes, heart attack, and asthma) using neural networks. Neural networks are ideal for problems that involve pattern recognition. The data for the U.S. (475,687 records) were collected from the Behavioral Risk Factor Surveillance System (BRFSS) database of the Centers for Disease Control and Prevention for the year 2012. Our study identifies strong trends in the association between certain chronic diseases and certain behavioral habits. Our finding of a portfolio of risk factors contributes to sustaining individual well-being and promoting public health.

The rest of the paper is organized as follow: Section 2 offers the background for the research; Section 3 defines the research methodology; Section 4 discusses the analyses and results; Section 5 offers the scope and limitations of the research; and Section 6 gives conclusions and policy implications with future research directions.

## 2. Research Background

### 2.1. Behavioral Factors and Chronic Diseases

Chronic, or non-communicable, diseases are those that progress slowly but have a long duration. They are not passed from person to person. Chronic diseases include cardiovascular diseases such as heart attacks and strokes, chronic respiratory diseases such as asthma, and diabetes. In the U.S., chronic diseases not only affect the quality of life; they also drive up healthcare costs and limit healthcare affordability, and they occupy an integral aspect of the economy. Most chronic diseases are preventable and can be mitigated. This research is focused on the predominant chronic conditions of heart attack and stroke, asthma, and diabetes.

Strategies and interventions for reducing risk factors that cause or worsen chronic conditions are extremely important. The U.S. Centers for Disease Control and Prevention (CDC) posits that elimination of the three risk factors of poor diet, smoking, and physical inactivity can eliminate a large percentage of heart attacks, strokes, and diabetes [1]. The CDC suggests a framework of four domains for chronic disease prevention efforts. Epidemiology and surveillance efforts include identification of vulnerable and affected populations, providing solutions, and monitoring the progress. Environmental approaches include facilitating and promoting healthy behaviors in various settings. Health system interventions include clinical and preventive efforts at improving healthcare delivery, reducing risk factors, and managing complications. And community programs include those linked to clinical services to promote effective management of chronic conditions. The domains represent strategies and interventions directed toward improving public health across a range of chronic diseases. Most research on chronic disease mitigation and prevention fall into one of the categories in the framework [6]. We categorize our study in the environmental approach to the management of chronic conditions. We identify unhealthy behavioral tendencies that influence chronic conditions and suggest efforts to cultivate healthy behaviors by individuals. The incidence of non-communicable chronic diseases is strongly associated with the globalization of unhealthy lifestyles [7,8,9], including improper nutrition, alcohol and tobacco overuse, lack of physical activity, environmental pollution, and chronic infection [10]. Physical inactivity is a major risk factor in cardiovascular diseases, such as type II diabetes, hypertension, anxiety, and depression [11], all of which are leading factors of morbidity and mortality [12,13]. Obesity, which may result from lack of exercise, increases the chances of chronic inflammation, insulin resistance, glucose intolerance, and hormonal imbalance [14,15,16]. A healthy diet, including a balanced intake of fruits and vegetables, is one of many measures to counter obesity and other conditions [17]. Smoking is another important risk factor for chronic diseases. The incidence and duration of smoking has been associated with an increased risk of chronic obstructive pulmonary disease [18]. In the U.S. in 2015, approximately 15% of all adults (36.5 million) were cigarette smokers, and more than 13 million live with a smoking-related disease [3]. Additionally, people diagnosed with smoking-related chronic diseases were found to be current smokers. There is a need for evidence-based approaches that prevent smoking initiation or increase smoking cessation in the U.S. The behavioral risk factors of smoking, alcohol consumption, improper diet, and lack of physical activity contribute to about half of the burden of diseases in developed countries [7,19]. These factors are not equally distributed through the population but tend to concentrate and affect the most vulnerable segments [20]. The exposure to behavioral risk factors is temporal and varies with demographic characteristics such as age and income, among others [21]. Also, behavioral habits occur on a long-term basis and can have an impact on the health of individuals [21]. Epidemiological studies emphasize the importance of measuring the impact of multiple lifestyle risk behaviors on people’s health [21].

Promoting good behavioral habits can positively influence the prevention or delay of disability, dementia, frailty, and non-communicable/chronic conditions [21]. Modifying behavioral habits consists of disrupting the cue-response association, the fundamental principle for habit formation [22]. Avoiding exposure to everyday cues can help facilitate behavior change. Our research on behavioral habits and the association with chronic conditions is based on the underlying premise that behavioral habits, if identified, can be addressed and modified.

### 2.2. Neural Networks in Healthcare

Healthcare is a domain that has deployed health analytics for various areas, including preventive health and wellness and disease management [23]. In disease management, by identifying the affected populations in different disease categories, analytics helps target customized management techniques and practices that will mitigate the disease as well as prevent the onset of associated medical conditions.

Because of their ability to perform input-output mapping of data without a priori knowledge of distribution patterns, neural networks are appropriate for applications that deal with large volumes of data and with fuzzy or noisy data. These networks have the ability to learn from experience, generalize from previous examples, and abstract relevant features from irrelevant data inputs [24]. Neural network applications in the domain of chronic disease management include automatic prediction of exacerbations in Chronic Obstructive Pulmonary Disorder [25]; diagnosing myocardial infarction [26,27,28,29], coronary artery disease [30,31,32], chronic heart failure [33]; predicting heart diseases [34]; classifying other types of heart disease [35]; diagnosing diabetes on small mobile devices [36]; and identifying behavioral health problems of patients who are at high risk for hospital admission [37]. In most chronic diseases, early detection is beneficial for effective management of the conditions.

### 2.3. The Neural Network Model

A neural network consists of a series of processing elements called neurons that are interlinked to form a network. Each link has a weight associated with it. Each neuron receives stimuli (information) from the surrounding neurons that are linked to it, processes the information, and produces an output [38]. A neural net consists of an input layer, one or more hidden layers, and the output layer. The neurons in the input layer receive stimulus from outside the network; the neurons in the hidden layer receive stimulus from the interconnected neurons and pass on the output to other neurons within the network; and the neurons in the output layer receive the stimulus from the linked neurons and pass on the output externally. Different neural network structures arise based on combinations of neurons and layers [39].

In this research, a Multilayer Perceptron (MLP) feed forward neural network was used and trained with the error back propagation algorithm. The MLP consists of an input layer, one or more hidden layers, and an output layer. Information moves in a forward direction through the network. The number of neurons at the input layer is guided by the number of independent variables, while the number of neurons at the output layer correlates with the number of values that need to be predicted. Unlike the input and output layers, there are no widely accepted rules for determining the optimal number of hidden layers. A less than optimal number of hidden units will result in hampering the network’s learning of the input-output mapping. A more than optimal number of hidden units will result in the network generalizing poorly on new data. The optimal configuration is most often derived by trial and error approach [24].

The network is initially fed an array of input-output values. It is then trained using the back propagation algorithm to assign appropriate weights for the connections and calculate the outputs. The accuracy of the predicted outputs is then estimated by comparing with known values. Error signals are created out of such comparisons and are propagated backwards through the various layers. The network then adjusts and updates the weights appropriately. These training iterations are repeated until the network learns to adjust the weights and arrives at predictions that show a minimal difference with the actual values.

## 3. Research Methodology

### 3.1. Data Collection

Data for 475,687 records were collected from the CDC’s Behavioral Risk Factor Surveillance System (BRFSS) database for the year 2012. The indicators for behavioral habits include alcohol consumption, regular soda consumption (sugar), frequency of smoking, frequency of drinking alcohol, weekly working hours, fruit consumption, vegetable consumption, and exercise. The indicators for chronic diseases include heart attack, stroke, asthma, and diabetes. The demographic variables of marital status, income level, and age are included. The data for the variables was extracted at a state level for the state of New York. The variables and their description are shown in Table 1.

For the neural network analysis, the independent variables were the behavioral habits of alcohol consumption, regular soda consumption (sugar), frequency of smoking, frequency of drinking alcohol, weekly working hours, fruits consumption, vegetables consumption, and exercise. The dependent variables were heart attack, stroke, asthma, and diabetes.

We analyzed the data for the following proposition. We have included demographic variables in the analysis. Even though the demographic variables are not modifiable, they play a major role in the onset of chronic conditions. Also, analyzing demographics in relation to chronic diseases can facilitate targeting and planning of future intervention and wellness programs.

Chronic diseases have a positive association with alcohol consumption, soda consumption, weekly working hours, marital status, income level, and age; and a negative association with fruit and vegetable consumption, and exercise.

### 3.2. Analytics Tool Selection

SPSS Modeler was utilized with its functions of Neural Networks, Association, and Bayesian networks. The model building stage consisted of experimenting with one and two hidden layers with various combinations of nodes to determine the best model. The training-testing percentages of 50–50, 60–40, and 70–30 were used. Neural Network builds the model by learning from the potential correlation between independent (behavioral habits) and dependent (chronic diseases) variables. It then validates the model results by comparing the predicted values with the actual values. In such applications, neural network systems are better than conventional computers that follow a set of instructions to solve a problem.

## 4. Analysis and Results

SPSS Modeler and Auto Classifier Model are used to analyze the dataset. The analyses for model building, training, and testing phases are described below.

### 4.1. Neural Network Training and Testing

Neural Network with Auto Classifier model was selected as the one that works best with noisy and fuzzy data. Independent variables were selected in accordance with the weights assigned by the model. We adopted different combinations of hidden layers (one and two) and nodes, and experimented with different partition rates of the data set for training and testing: 50–50, 60–40, and 70–30 (training-testing %). Since we have a comparatively large dataset, we had the option of adopting the most strict partition rate for the neural net. The logic is that if the model functions well under such strict conditions, it would illustrate that the association is explicit and solid.

The iterations of 50%, 40% and 30% of the data set to test the training results for prediction were adopted to represent strict, moderate, and loose conditions, respectively. The Auto Classifier model was used to explore possible classification models other than Neural Network for similar predictions using different approaches. The aggregate results are compared to determine the best approach.

**Figure 1:**
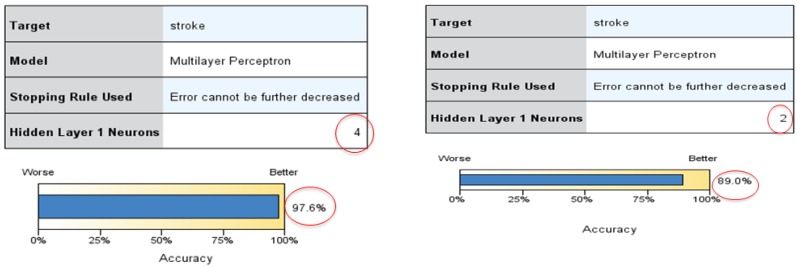
The best Neural Network models with data sizes 2907 and 550.

We set the chronic disease of stroke and heart attack as the target or dependent variables, and all other behavioral habits variables as the predictor/independent (input) variables. Neural network models were run separately for each dependent variable. The six most important predictors for each of the dependent variables were selected to run the models again. The data had 2907 rows for analysis. Figure 1 shows the best model for predicting stroke, with the highest accuracy of 97.6%. The best fit model has one input layer, one hidden layer with four nodes, and one output layer. The partition rate of 50–50 was used.

The top three predictors for stroke under this model are age, weekly working hours, and frequency of drinking soda (sugar). Age is the number one predictor of stroke: the older the person, the higher the possibility of a stroke. Similarly, the higher the weekly working hours and the higher the frequency of drinking soda, the higher the possibility of having a stroke. The other predictors for stroke, in order, are consumption of vegetables and fruits, consumption of alcohol, income, frequency of smoking, and frequency of exercise. It has to be noted that in the data set, there was a disparity in the number of people who were diagnosed with a stroke (50 records) when compared to those who were never diagnosed with a stroke (2857 records). Given this situation, the model would have been able to precisely predict only one of the two groups, namely the group that was never diagnosed with a stroke. To solve this problem, the data size of people who were never diagnosed with a stroke was reduced to 500. Using different training/testing percentages, the model with data size 550 was selected as the one with the highest accuracy (89.1%). This model is also shown in Figure 1.

The top three predictors for stroke with this model are working hours, marital status, and consumption of fruits. The model classified 42.3% of people who were diagnosed with a stroke and 100% of people who were never diagnosed with a stroke. For the training data, the model predicted 40.741% of people diagnosed with a stroke, and 94.094% of people who were never diagnosed with a stroke. The prediction accuracy in training was higher than in testing. A summary of our analyses using neural network is shown in Table 2 with the best models highlighted.

The top three predictors for stroke with this model are working hours, marital status, and consumption of fruits. The model classified 42.3% of people who were diagnosed with a stroke and 100% of people who were never diagnosed with a stroke. For the training data, the model predicted 40.741% of people diagnosed with a stroke, and 94.094% of people who were never diagnosed with a stroke. The prediction accuracy in training was higher than in testing. A summary of our analyses using neural network is shown in Table 2 with the best models highlighted.

### 4.2. Comparison with Other Models

#### 4.2.1. Association

**Figure 2:**
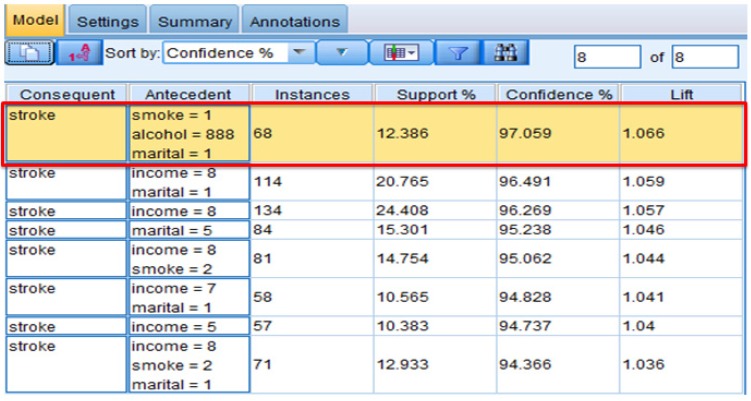
Association-interpreting the results.

The threshold (minimum confidence) was set to 94% in keeping with the high accuracy requirement for healthcare analytics. There were 68 people (12.386% of all records) who were married, did not drink in the past 30 days, and had smoked at least 100 cigarettes in their entire life. Of this group, 97.059% were diagnosed with a stroke. There were 114 married people whose income is more than $75,000. Of this group, 96.5% were diagnosed with a stroke. There were 134 people whose income is more than $75,000. Of this group, 96.4% were diagnosed with a stroke. The important predictors for stroke using association are marital status, alcohol consumption, smoking, and income. The results are shown in Figure 2.

#### 4.2.2. Bayesian Networks

**Figure 3:**
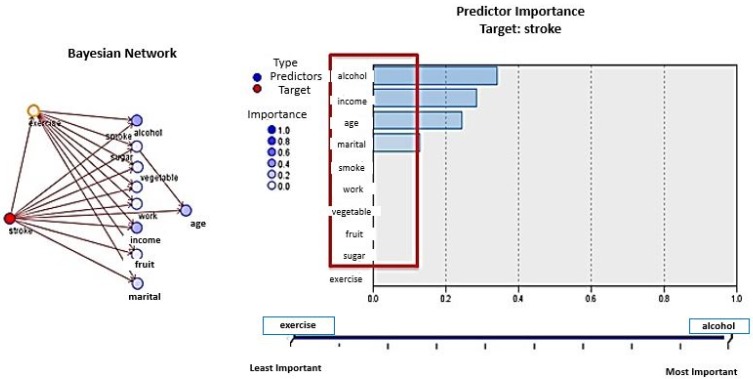
Bayesian Networks: Model summary and predictor importance.

Analysis using Bayesian networks shows that the important predictors are alcohol, income, age, and marital status. The connections between exercise and other variables do not indicate causality but rather conditional dependencies or interrelatedness. Most of the people who did not have a stroke were those who exercised regularly and who did not smoke more than 100 cigarettes in their entire lives. The model is 96.09% accurate in the training phase. The model was less accurate in predicting patients diagnosed with a stroke (76.67%) than in predicting patients who were never diagnosed with a stroke (98.4%). The model did not do well in the testing phase (70.9%) (Figure 3).

In summary, we show that behavioral habits such as physical inactivity and smoking make a significant contribution to the incidence of chronic diseases. People with higher income and with long working hours are more likely to be diagnosed with chronic diseases such as stroke. In comparing various data mining techniques, we see that in a biased dataset, only the Bayesian network model worked well because the majority of the predictors in the project were categorical. Neural network and association techniques could predict only one of the two groups well (in this case, those who were never diagnosed with stroke). After adjusting to an unbiased dataset, all the predictive techniques worked well.

## 5. Scope and Limitations

Our research does have some limitations. First, our study is cross-sectional and covers the year 2012, while other studies could cover a larger time span. However, the research has value in that behavioral habits may occur on a long-term basis and therefore have an impact on the health of individuals. Second, the data is extracted at a state level (New York) thereby limiting the generalizability of the results. Future studies may be conducted at a more comprehensive national or global level, with more extensive coverage of chronic conditions. Third, it is possible that there are other variables that better explain the phenomenon of behavioral habits and chronic conditions. Fourth, it is also possible for the data to be skewed, thereby impacting the results. Lastly, the current research used SPSS Modeler, which is one of many analytic tools that are available and can be effectively deployed for data analyses.

## 6. Conclusions and Policy Implications

Our research shows that behavioral habits such as physical activity, alcohol consumption, and smoking are significant contributors to chronic diseases. In the investigation of chronic diseases most studies analyze individual behavioral habits such as diet [14,15,17], physical activity [16,17,40], smoking [18,41], and alcohol consumption [42,43]. In contrast, our research uses neural networks to analyze the combined influence of multiple behavioral habits on chronic diseases. We add to the body of literature on chronic diseases by offering a portfolio of behavioral risk factors. Neural networks have the advantage over other programs in analyzing large and complex data sets relating to the promotion of wellness and disease management [23]. Identification of populations of patients in different behavioral risk categories can improve delivery of wellness programs relating to chronic diseases. As an example, identifying patients who are at a risk of developing diabetes can help the design of prevention and mitigation programs that not only prevent the occurrence of diabetes but also of other related chronic conditions [23].

Physical activity is important in reducing the rates of cardiovascular disease and other chronic conditions. An active physical lifestyle will contribute twofold: by reducing mortality and promoting healthy cognitive and psychological well-being. Even people with certain chronic conditions can reduce the risk of premature mortality by increasing their physical activity to moderate levels [40].

We show that alcohol consumption is another important indicator of chronic diseases. The International Classification of Diseases (ICD) published by the World Health Organization provides a system of diagnostic codes for classifying diseases. According to this, twenty five chronic disease and condition codes are attributable to alcohol [42,43], thus highlighting the criticality of alcohol consumption in mortality and morbidity.

Our findings on smoking as an indicator are significant. Smoking causes a systemic oxidant-antioxidant imbalance and an inflammatory response, both of which increase vulnerability to chronic conditions [41]. Dietary consumption of fruits and vegetables is an important component as a daily source of nutrition, dietary fiber, and phytochemicals [44]. A balanced diet of fruits and vegetables indirectly influences chronic diseases, by preventing weight gain and obesity, both of which are leading causes for chronic diseases such as type-2 diabetes [44]. It also reduces symptoms such as chronic inflammation, glucose intolerance and balanced hormone metabolism [14,17], all of which are precursors for chronic diseases.

Also relevant are socio-economic factors such as income and working hours. People with higher income and with longer weekly working hours are more likely to be diagnosed with chronic conditions like stroke. Regularly working long hours over a period of time is significantly associated with heightened risks of heart disease [45], hypertension [46], arthritis, diabetes [47] and non-skin cancer.

This study also shows that in a biased dataset, only the Bayesian network model worked well because the majority of predictor variables are categorical. After adjusting the data to make it unbiased, all the predictive techniques including neural network and association worked well.

Our study has several policy implications. First, our research focuses on the prevention and mitigation of chronic diseases, which is a top-level national objective from a healthcare and economic perspective. From a healthcare perspective, it addresses improving the public health of the population; and from an economic perspective, it addresses lifting the burden of the escalating cost of chronic disease management in the national healthcare budget. Our results have implications for preventive healthcare for chronic diseases at an individual and national level. At the individual level, identification of a portfolio of risk factors enables people to make lifestyle changes aimed at countering chronic diseases. At the national level, the portfolio enables governments to frame campaigns and policies that promote healthy behavioral habits to mitigate/prevent chronic diseases and promote public health.

Some campaigns are already underway in encouraging healthy behavioral habits. The Child Nutrition and WIC Reauthorization Act of 2004 was passed to encourage schools to offer wellness policies aimed at offering healthy nutrition in lunchrooms [48]. On the diabetes front, the “Managing Diabetes at School Playbook” campaign includes measures undertaken by the CDC to educate schoolteachers and staff in managing diabetes in schoolchildren [3]. On the obesity front, the “*Let’s Move!*” campaign initiated by former First Lady Michelle Obama is about educating parents to foster an environment that supports healthy choices for their children and themselves, providing healthier foods at schools and helping children become more physically active [49]. With regard to smoking, the CDC’s national “Tips from Former Smokers” campaign was initiated in 2012 with the objective of educating people about the harmful effects of smoking by featuring people who are living with serious long-term health effects of smoking and second-hand smoke exposure [50]. The Food and Drug Administration’s “Real Cost” campaign aims to make youth aged 12–17 aware of the risks of smoking by advertising the consequences that teens are most concerned about, such as loss of control due to addiction, loss of teeth, and damage to skin [50].

In addition to these initiatives, national policies regulating working hours are needed to reduce stress and improve health of employees, with the added benefit to employers of improved productivity resulting from satisfied employees [51,52].

In terms of future research, the phenomenon of chronic diseases is large enough to warrant future studies that encompass a more expansive dataset and a varied set of analytic techniques. In addition to association, future research can explore causality between behavioral habits and chronic diseases. Also of interest is the exploration of the role of gender in the association between working hours and chronic conditions. For instance, are women more susceptible than men to chronic diseases with prolonged exposure to longer working hours?

It is important to consider the influence of demographic factors—such as age, gender, body weight, and education—on chronic conditions. For alcohol-related studies, the drinking culture, alcohol policy, drinking environment, the association between smoking and chronic conditions, and other societal factors, should be looked at closely.

There is a pressing need for studies that deploy novel integrative approaches. Certain chronic diseases such as type 2 diabetes and atherosclerosis have an inflammation component that is “pathophysiological.” That is, the cause for the inflammation is not only physiological but also pathological (involving the mind) [53]. Therapeutic treatment of the inflammatory response is now being considered to manage the inflammation and investigate the causes. In the future there is scope for such translational research, integrating several disciplines, resources, expertise, and techniques to promote enhancements in prevention, diagnosis, and therapies for chronic diseases.

Lifestyle medicine is a fairly recent holistic approach to management of chronic diseases [54]. Lifestyle medicine addresses diet, physical activity, behavioral change, body weight control, tobacco and substance abuse, stress management, spirituality, and mind and body techniques. Future studies can investigate this approach and explore associated challenges and issues in the management of chronic conditions.

In today’s digitized world, social networks are increasingly used to build relationships with patients and families in order to extend patient care into the arenas of home, school and community [55]. Such relationships help integrate behavioral health and the social context of patient care into chronic disease management.

The future for chronic disease management is in revolutionizing healthcare by the introduction of healthcare information technology (HIT) in identifying diseases, personalizing treatment protocols with digital and web-based technologies, and integrating patient symptoms and medication data with environmental and genomic data [56]. The future also incorporates utilization of novel treatment approaches that combine psychological well-being with physiological well-being.

## Figures and Tables

**Table 1:** Variables in the research.

Variable	Description of Variables
**Behavioral Habits:**	
Smoking history	Smoked at least 100 cigarettes in the entire life or not
Frequency of drinking alcohol	Number of days of having at least one alcoholic drink per week or per month during the past 30 days
Frequency of drinking soda (sugar)	Frequency of drinking regular soda during the last 30 days:
	1__ - Times per day (00–99)
	2__ - Times per week (00–99)
	3__ - Times per month (00–99)
Frequency of eating fruits	Times per day, week, or month eating fruit (not counting juice):
	1__ - Times per day (00–99)
	2__ - Times per week (00–99)
	3__ - Times per month (00–99)
Frequency of eating vegetables	Times per day, week, or month eating vegetables (include tomatoes, tomato juice or V-8 juice, corn, eggplant, peas, lettuce, cabbage, and white potatoes that are not fried such as baked or mashed potatoes):
	1__ - Times per day (00–99)
	2__ - Times per week (00–99)
	3__ - Times per month (00–99)
Exercise	Participated in any physical activity or exercise, other than a regular job, such as running, calisthenics, golf, gardening, or walking
**Chronic Diseases:**	
Heart attack	If the person had a heart attack
Stroke	If the person had a stroke
Asthma	If the person had an asthma attack
Diabetes	If the person had diabetes
Weekly working hours	Hours working per week at all jobs and businesses combined
**Demographics:**	
Marital status	Married, Divorced, Widowed, Separated, Never married, a member of an unmarried couple (1–6)
Income level	Annual household income level
Age	Age of the person

**Table 2:** Summary of Neural Network analyses.

Chronic Disease	Input	Output	Training	Testing	Hidden Layers	Nodes	Accuracy	Top 3 Predictor Importance
**Data Size 2907**								
Heart Attack	10	1	50	50	1	Auto 5	95.0	age, work, fruit
	10	1	70	30	1	Auto 2	96.0	age, vegetable, work
	10	1	60	40	1	Auto 3	95.1	age, fruit, alcohol
	10	1	50	50	2	(3,8)	95.0	age, vegetable, income
	10	1	50	50	1	6	95.0	age, fruit, sugar
	10	1	50	50	2	(4,5)	95.0	age, sugar, work
Stroke	10	1	50	50	1	Auto 1	96.8	Marital, sugar, fruit
	10	1	70	30	1	Auto 4	97.6	Age, work sugar
	10	1	60	40	1	Auto 2	96.6	Age, marital, sugar
	10	1	50	50	1	9	96.8	Sugar, work, fruit
	10	1	50	50	2	(2,9)	96.8	Age, work alcohol
	10	1	50	50	2	(9,9)	96.8	Smoke, fruit, work
	10	1	50	50	2	(3,3)	96.8	Age, work, income
**Data Size 550**								
Stroke	10	1	50	50	1	Auto 2	89.0	Work, marital fruit
	10	1	70	30	1	Auto 2	89.1	Vegetable, marital, work
	10	1	60	40	1	Auto 3	87.7	Age, marital, work
	10	1	50	50	1	9	85.1	Fruit, vegetable, sugar
	10	1	50	50	2	(3,3)	86.8	Work, vegetable, income

## References

[1] Centers for Disease Control and Prevention National Center for Chronic Disease Prevention and Health Promotion 2010. http://www.cdc.gov/chronicdisease/overview/index.htm.

[2] Bodenheimer T., Chen E., Bennett H.D. (2009). Confronting the growing burden of chronic disease: Can the U.S. health care workforce do the job?. Health Aff..

[3] Centers for Disease Control and Prevention National Center for Chronic Disease Prevention and Health Promotion 2016. http://www.cdc.gov/chronicdisease.

[4] Harris J.R., Wallace R.B. (2012). The institute of medicine’s new report on living well with chronic illness. Prev. Chronic Dis..

[5] Anderson G., Horvath J. (2002). The growing burden of chronic disease in America. Public Health Rep..

[6] Centers for Disease Control and Prevention The Four Domains of Chronic Disease Prevention. http://www.cdc.gov/chronicdisease/resources/publications/four-domains.htm.

[7] World Health Organization The World Health Report 2002: Reducing Risks, Promoting Healthy Life. http://www.who.int/whr/2002/en/.

[8] World Health Organization Global Strategy on Diet, Physical Activity and Health. http://www.who.int/dietphysicalactivity/en/.

[9] NCD Alliance NCD Alliance Activity Report 2015: Advocacy Milestones, Changing Gears, Listening and Convening. https://ncdalliance.org/news-events/blog/ncd-alliance-activity-report-2015-advocacy-milestones-changing-gears-listening-convening.

[10] Bonita R., Beaglehole R., Kjellström T. (2006). Basic Epidemiology.

[11] Warburton D.E., Nicol C.W., Bredin S.S. (2006). Health benefits of physical activity: The evidence. Can. Med. Assoc. J..

[12] World Health Organization (2016). Cardiovascular diseases (CVDs): Fact Sheet No. 317. http://www.who.int/mediacentre/factsheets/fs317/en/.

[13] Li J., Siegrist J. (2012). Physical activity and risk of cardiovascular disease—A meta-analysis of prospective cohort studies. Int. J. Environ. Res. Public Health.

[14] Bhupathiraju S.N., Tucker K.L. (2011). Greater variety in fruit and vegetable intake is associated with lower inflammation in Puerto Rican adults. Am. J. Clin. Nutr..

[15] Dossus L., Kaaks R. (2008). Nutrition, metabolic factors and cancer risk. Best Pract. Res. Clin. Endocrinol. Metab..

[16] Hursting S.D., Lashinger L.M., Wheatley K.W., Rogers C.J., Colbert L.H., Nunez N.P., Perkins S.N. (2008). Reducing the weight of cancer: Mechanistic targets for breaking the obesity—Carcinogenesis link. Best Pract. Res. Clin. Endocrinol. Metab..

[17] Calder P.C., Ahluwalia N., Brouns F., Buetler T., Clement K., Cunningham K., Esposito K., Jönsson L.S., Kolb H., Lansink M. (2011). Dietary factors and low grade inflammation in relation to overweight and obesity dietary factors and low grade inflammation in relation to the metabolic syndrome. Br. J. Nutr..

[18] Taghizadeh N., Vonk J.M., Boezen H.M. (2016). Lifetime smoking history and cause-specific mortality in a cohort study with 43 years of follow-up. PLoS ONE.

[19] Lim S.S., Vos T., Flaxman A.D., Danaei G., Shibuya K., Adair-Rohani H., AlMazroa M.A., Amann M., Anderson H.R., Andrews K.G. (2013). A comparative risk assessment of burden of disease and injury attributable to 67 risk factors and risk factor clusters in 21 regions, 1990–2010: A systematic analysis for the global burden of disease study 2010. Lancet.

[20] Blane D., Marmot M., Wilkinson R.G. (2006). The Life Course, the Social Gradient and Health. Social Determinants of Health.

[21] Lafortune L., Martin S., Kelly S., Kuhn I., Remes O., Cowan A., Brayne C. (2016). Behavioural risk factors in mid-life associated with successful ageing, disability, dementia and frailty in later life: A rapid systematic review. PLoS ONE.

[22] Verplanken B., Wood W. (2006). Interventions to break and create consumer habits. J. Public Policy Mark..

[23] Raghupathi W., Raghupathi V. (2013). An overview of health analytics. J. Health Med. Inform..

[24] Using analytics and collaboration to improve healthcare quality and outcomes. http://resources.information-management.com/content50200.

[25] Fernández-Granero M.A., Sánchez-Morillo D., León-Jiménez A., Crespo L.F. (2014). Automatic prediction of chronic obstructive pulmonary disease exacerbations through home telemonitoring of symptoms. Biomed. Mater. Eng..

[26] Baxt W.G., Shofer F.S., Sites F.D., Hollander J.E. (1991). A neural computational aid to the diagnosis of acute myocardial infarction. Ann. Intern. Med..

[27] Eggers K.M., Ellenius J., Dellborg M., Groth T., Oldgren J., Swahn E., Lindahl B. (2007). Artificial neural network algorithms for early diagnosis of acute myocardial infarction and prediction of infarct size in chest pain patients. Int. J. Cardiol..

[28] Ellenius J., Groth T., Lindahl B., Wallentin L. (1997). Early assessment of patients with suspected acute myocardial infarction by biochemical monitoring and neural networks analysis. Clin. Chem..

[29] Pedersen S.M., Joegensen J.S., Pedersen J.B. (1996). Use of neural networks to diagnose acute myocardial infarction II, A clinical application. Clin. Chem..

[30] Lewenstein K. (2001). Radial basis function neural network approach for the diagnosis of Coronary artery disease based on the standard ECG exercise test. Med. Biol. Eng. Comput..

[31] Mobley B.A., Schechter E., Moore W.E., Mckee P.A., Eichner J.E. (2000). Predictions of coronary stenosis by artificial neural network. Artif. Intell. Med..

[32] Shen Z., Clarke M., Jones R., Alberti T. (1995). A new neural network structure for detection of coronary heart disease. Neural Comput. Appl..

[33] Leslie S.J., Hartswood M., Meurig C., McKee S.P., Slack R., Procter R., Denvir M.A. (2006). Clinical decision support software for management of chronic heart failure: Development and evaluation. Comput. Biol. Med..

[34] Carlucci D., Renna P., Schiuma G. (2013). Evaluating service quality dimensions as antecedents to outpatient satisfaction using back propagation neural network. Health Care Manag. Sci..

[35] Yan H., Jiang J., Zheng J., Peng C., Li Q. (2006). A multilayer perceptron based medical decision support system for heart disease diagnosis. Expert Syst. Appl..

[36] Xu M., Wong T.C., Chin K.S. (2013). Modeling daily patient arrivals at Emergency Department and quantifying the relative importance of contributing variables using artificial neural network. Decis. Support Syst..

[37] Bates D.W., Saria S., Ohno-Machado L., Shah A., Escobar G. (2014). Big data in healthcare: Using analytics to identify and manage high-risk and high-cost patients. Health Affair..

[38] Karan O., Bayraktar C., Gumuskaya H., Karlık B. (2012). Diagnosing diabetes using neural networks on small mobile devices. Expert Syst. Appl..

[39] Raghupathi V., Raghupathi W. (2015). A neural network analysis of treatment quality and efficiency of hospitals. J. Health Med. Inform..

[40] McKinney J., Lithwick D.J., Morrison B.N., Nazzari H., Isserow S.H., Heilbron B., Krah A.D. (2016). The Health Benefits of Physical Activity and Cardiorespiratory Fitness. BC Med. J..

[41] Yanbaeva D.G., Dentener M.A., Creutzberg E.C., Wesseling G., Wouters E.F. (2007). Systemic effects of smoking. Chest.

[42] Rehm J., Baliunas D., Borges G.L.G., Graham K., Irving H., Kehoe T., Parry C.D., Patra J., Popova S., Poznyak V. (2010). The relation between different dimensions of alcohol consumption and burden of disease: An overview. Addiction.

[43] Shield K.D., Parry C., Rehm J. (2013). Focus on: Chronic diseases and conditions related to alcohol use. Alcohol Res. Curr. Rev..

[44] Boeing H., Bechthold A., Bub A., Ellinger S., Haller D., Kroke A., Leschik-Bonnet E., Müller M.J., Oberritter H., Schulze M. (2012). Critical review: Vegetables and fruit in the prevention of chronic diseases. Eur. J. Nutr..

[45] Virtanen M., Heikkila K., Jokela M., Ferrie J.E., Batty G.D., Vahtera J., Kivimäki M. (2012). Long working hours and coronary heart disease: A systematic review and meta-analysis. Am. J. Epidemiol..

[46] Yang H., Schnall P.L., Jauregui M., Su T.-C., Baker D. (2006). Work hours and self-reported hypertension among working people in California. Hypertension.

[47] Kawakami N., Araki S., Takatsuka N., Shimizu H., Ishibashi H. (1999). Overtime, psychosocial working conditions, and occurrence of non-insulin dependent diabetes mellitus in Japanese men. J. Epidemiol. Commun. Health.

[48] Serrano E., Kowaleska A., Hosig K. (2007). Status and goals of local school wellness policies in virginia: A response to the child nutrition and WIC reauthorization act of 2004. J. Nutr. Educ. Behav..

[49] Let’s Move!. http://www.letsmove.gov/.

[50] Health and Human Services Be Tobacco Free. http://betobaccofree.hhs.gov/campaigns/.

[51] Butler A.B., Grzywacz J.G., Ettner S.L., Liu B. (2009). Workplace flexibility, self-reported health, and health care utilization. Work Stress.

[52] Grzywacz J.G., Carlson D.S., Shulkin S. (2008). Schedule flexibility and stress: Linking formal flexible arrangements and perceived flexibility to employee health. Community Work Fam..

[53] Tabas I., Glass C.K. (2013). Anti-Inflammatory therapy in chronic disease: Challenges and opportunities. Science.

[54] Kushner R.F., Sorensen K.W. (2013). Lifestyle medicine: The future of chronic disease management. Curr. Opin. Endocrinol..

[55] Siwicki B. Are Social Networks the Future of Chronic Disease Care?. http://www.healthcareitnews.com/news/are-social-networks-future-chronic-disease-care.

[56] Himes B.E., Weitzman E.R. (2016). Innovations in health information technologies for chronic pulmonary diseases. Respir. Res..

